# Impact of catheter positioning on planned target volume (PTV) coverage and organs at risk (OARs) in high-dose-rate (HDR) endotracheal brachytherapy: a comparative 2D vs 3D study

**DOI:** 10.7150/ijms.136860

**Published:** 2026-09-24

**Authors:** J Navarro Castellón, I Flores Cacho, MC López Carrizosa, C Ibáñez Villoslada, M Domínguez Morcillo, E Carrasco esteban, P Sáez Bueno, Z Zalabarría Zarrabeitia, A Pontes Sanz, N Curiel Pérez, FJ San Miguel Avedillo, M Cámara Gallego, MA Ortega, MA Sáez García

**Affiliations:** 1 Department of Radiation Oncology, Hospital Central de la Defensa Gómez Ulla, CSVE.; 2 Department of Radiation Protection and Medical Physics, Hospital Central de la Defensa Gómez Ulla, CSVE.; 3 Department of Medicine and Medical Specialities, Faculty of Medicine and Health Sciences, Network Biomedical Research Center for Liver and Digestive Diseases (CIBEREHD), University of Alcalá, 28801 Alcala de Henares, Spain.; 4 Ramón y Cajal Institute of Sanitary Research (IRYCIS), 28034 Madrid, Spain.; 5 Department of Pathology, Hospital Central de la Defensa Gómez Ulla, CSVE.

**Keywords:** endobronchial brachytherapy, HDR, 2D planning, 3D planning

## Abstract

**Background and Purpose:**

High-dose-rate endobronchial brachytherapy (HDR-BTE) is an effective technique for the treatment of malignant tracheobronchial tumors. Although two-dimensional (2D) planning provides limited dosimetric information and does not accurately assess the relationship between the catheter and the tumor or the dose delivered to organs at risk (OARs), it continues to be used because it ensures adequate PTV coverage, is associated with low toxicity, and minimizes the risk of catheter displacement by avoiding patient transfer to the CT simulation room.

**Patient and Methods:**

An 83-year-old patient with 2D HDR-BTE for tracheal recurrence of previously resected lung adenocarcinoma was analyzed and compared with four simulated 3D plans based on catheter position relative to the tumor volume, while keeping the active length and prescribed dose constant.

**Results:**

The favorable oblique scenario achieved the best PTV coverage (D90: 9.05 Gy; V100: 100%), although with highest V150 (96.47%) and homogeneity index (IH: 5.14). The unfavorable oblique and posterior positions significantly reduced coverage (V100: 51.4% and 61.29%, respectively). The esophagus was the most sensitive organ, showing the highest doses in the posterior orientation of the catheter. The contralateral lung and heart received low doses; while the aorta showed higher doses in the favorable oblique scenario due to the location of the PTV.

**Conclusions:**

Catheter orientation in HDR-BTE has a significant impact on PTV coverage and the dose delivered to critical organs. Despite the trend toward 3D planning, there is no direct clinical evidence comparing the two techniques.

## Introduction

Endobronchial brachytherapy^1^ has become an established radiotherapy modality for palliative management, and in selected cases with curative intent, of bronchial obstruction caused by malignant tumors, as it offers the advantage of delivering very high doses directly to the lesion with minimal irradiation of organs at risk, either as a standalone treatment or in combination with external beam radiotherapy and/or chemotherapy. It alleviates associated symptoms such as cough, dyspnea, and hemoptysis, and a significant regression of lesions is frequently observed after treatment.

There is considerable variability in treatment regimens and in the dose delivered per fraction (between 5 and 10 Gy), and a standardized protocol has not yet been established. The total dose must be adjusted according to the treatment goal and intent, as well as the potential combination with external beam radiotherapy. A balance is sought between reducing the number of sessions (each requiring bronchoscopy with sedation) and limiting the dose per fraction to minimize toxicity.

The comparison between conventional two-dimensional (2D) and three-dimensional (3D) planning in HDR endobronchial brachytherapy (HDR-BTE) is essential to understand the strengths and limitations of each technique and, consequently, to optimize the selection of the most appropriate method depending on available resources and the clinical context^2^.

2D planning^3^ of HDR-BTE is based on the acquisition of orthogonal radiographs (anteroposterior and lateral) using a portable X-ray unit. From these images, it is possible to determine the position of the endobronchial catheter and, through geometric reconstruction, estimate dose distribution. It is a fast and simple procedure that does not require transferring the patient out of the radiosurgical suite and does not require advanced technological equipment. From a dosimetric perspective, 2D planning generally provides adequate coverage of the treatment volume. However, it has relevant limitations: the lack of three-dimensional anatomical information hinders precise delineation of the tumor volume, which necessitates the use of wider safety margins compared to CT-based planning. The latter represents a significant advancement, as it allows accurate reconstruction of catheter position, precise delineation of tumor volume (GTV/CTV), and identification of organs at risk (OARs), enabling calculation and optimization of the dose delivered both to the tumor and to these organs.

Although 3D planning^4^ using CT imaging provides greater anatomical accuracy, improved tumor delineation, and the possibility of calculating dose to OARs, the relevance of 2D planning should not be underestimated. It is a widely used technique that, for decades, has demonstrated effectiveness in terms of treatment volume coverage, local disease control, and improvement of obstructive symptoms (cough, dyspnea, and hemoptysis), with a low rate of complications and treatment-related side effects^5^.

The aim of this study is to perform a dosimetric comparison between a clinical 2D plan and simulated 3D plans under different scenarios, depending on catheter placement relative to the target volume and its proximity to organs at risk.

## Patients and Methods

### Clinical case

An 83-year-old male, former smoker (10 pack-years), with a medical history of chronic atrial fibrillation on acenocoumarol therapy, diabetes mellitus, and dyslipidemia, was diagnosed with lung adenocarcinoma pT3 pN0 M0^6^, negative for ALK, ROS1, and PDL1, and EGFR wild-type. He was initially treated with right lower lobectomy.

Three years after surgery, following episodes of cough and hemoptysis, a pedunculated lesion was identified on bronchoscopy in the middle third of the trachea, occupying 40-50% of the lumen and extending over 1 cm. Biopsy confirmed tracheal recurrence of lung adenocarcinoma. PET-CT revealed the lesion on the left lateral wall of the middle third of the trachea, at the level of the aortic arch, although it was not metabolically characterizable due to its small size. After multidisciplinary tumor board discussion, endoscopic treatment with argon plasma resection^7^, was performed, followed by high-dose-rate endobronchial brachytherapy^8^,^9^.

### Endobronchial brachytherapy technique

#### Implant placement

After appropriate clinical evaluation (including medical history review, diagnostic endoscopic examination, pathology report, and imaging studies), hospital admission was scheduled on the same day as the procedure.

The procedure was performed in a dedicated operating room under conscious sedation and cardiorespiratory monitoring. Using endoscopic visualization, the lesion was located 10 cm from the vocal cords and 5 cm from the main carina, and its macroscopic extent was determined to be 10 mm (measured longitudinally). Through the working channel of the bronchoscope, a bronchial catheter was introduced with a rigid guidewire inside to prevent kinking, positioning it in the middle third of the trachea under fluoroscopic guidance and endoscopic visualization. The rigid guidewire was then replaced with a simulation probe (graduated guidewire) for subsequent dosimetric planning.

Dosimetric planning was performed using the Oncentra Brachy® software, and treatment was delivered using an automatic afterloading system with Iridium-192 (MicroSelectron HDR®).

A fractionation scheme of 500 cGy per session was used, with 6 weekly applications, reaching a total dose of 30 Gy^10^.

### 2D clinical planning

#### Image acquisition

After catheter placement, images were acquired for treatment planning. A brachytherapy arc was positioned on the operating table, and two orthogonal radiographs were obtained using a portable X-ray unit: anteroposterior (source-to-arc distance of 59.2 cm) and lateral (49 cm).

Once obtained, it was confirmed that the radiographic films were centered on the reference points of the arc for geometric reconstruction, and that the simulation source positions were clearly visible to define the irradiation area and estimate the active treatment length.

#### Target volume definition

In the actual treatment, the radiation oncologist defined the target volume, encompassing the tumor plus a craniocaudal safety margin, for a total treatment length of 4 cm.

As this was a 2D plan, organs at risk (OARs) were not explicitly delineated; therefore, dose estimation to these structures was not possible.

For comparative analysis with 3D planning, and given that 2D planning does not allow direct extraction of dosimetric parameters, it was assumed that the catheter was centered within the tracheal lumen, in order to calculate dosimetric values and compare them across different scenarios.

#### Dose prescription and DVH evaluation

The dose was prescribed at a distance of 1 cm from the applicator, and an active length of 4 cm was established, corresponding to the tumor extension plus the longitudinal safety margin. It was ensured that the active length was covered by at least 90% of the prescribed dose.

#### Treatment

After completion of planning, treatment parameters were transferred to the afterloading system console for delivery. Treatment duration was very short (less than 3 minutes in all sessions), after connecting the implanted catheter to the high-dose-rate radiation source.

During treatment delivery, the patient's vital signs (heart rate and oxygen saturation) were continuously monitored. The patient was also observed via video surveillance, and an ionization chamber was used to detect potential radiation leakage.

### Simulation of three-dimensional (3D) planning in different scenarios

#### Image acquisition

Chest CT images from the same patient, with a slice thickness of 5 mm, were used to generate four different 3D plans using multiplanar and volumetric reconstructions, depending on catheter position relative to the lesion:

A. Centered: the catheter is centered within the tracheal lumen, analogous to the assumption in 2D planning.

B. Favorable oblique: the catheter is positioned close to the tumor.

C. Unfavorable oblique: the catheter is positioned away from the tumor.

D. Posterior: the catheter is located in the posterior tracheal wall, closer to the esophagus.

#### Target volume definition

Volume delineation followed GEC-ESTRO guidelines^11^, including the macroscopic tumor and the tracheal wall, with an additional longitudinal safety margin of 2 cm to define the Planning Target Volume (PTV).

Organs at risk^12^ included: heart, aorta, spinal cord, esophagus, and both lungs.

#### Planning

Once OARs and the PTV were contoured, planning was performed according to GEC-ESTRO recommendations. The catheter (hypothetical in this case, according to the four scenarios) was reconstructed, and dose was prescribed, optimized, and normalized to points located 1 cm from the catheter.

### Dosimetric evaluation

For the target volume (PTV), the following parameters were evaluated: D90, D98, V100, V150, and the homogeneity index (HI), defined as:



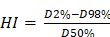



PFor OARs, the evaluated parameters were D0.035cc, D2%, D50%, and D98%, representing the maximum point dose, near-maximum dose (to 2% of the volume), mean dose, and minimum relevant dose, respectively.

Subsequently, a descriptive comparison between the plans was performed.

## Results

A comparative dosimetric analysis was performed between a real HDR endotracheal brachytherapy treatment planned in 2D and four simulated 3D dosimetric plans corresponding to the same clinical case.

### Dosimetric comparison between clinical 2D planning and 3D planning

**Table 1** summarizes the general planning parameters, showing that all scenarios maintained constant active length and prescription dose.

### Target volume coverage

PTV dosimetric parameters are presented in Table 2, showing relevant differences in dose coverage and distribution between the centered 2D plan and the different simulated 3D scenarios.

In terms of PTV coverage, the favorable oblique scenario showed the highest values of D90 (9.05 Gy) and D98 (6.95 Gy), achieving a V100 of 100%. The centered plan showed intermediate values (D90: 5.96 Gy; D98: 5.39 Gy), with nearly complete PTV coverage (V100: 99.63%). In contrast, scenarios where the catheter was positioned in an unfavorable oblique or posterior configuration showed a significant reduction in coverage, with D90 values of 4.09 Gy and 3.78 Gy, respectively, and V100 values of 51.4% and 61.29%.

Regarding high-dose regions, the volume receiving at least 150% of the prescribed dose (V150) was considerably higher in the favorable oblique plan (96.47%), compared with the centered plan (50.87%), posterior (22.23%), and unfavorable oblique (2.11%).

The homogeneity index (HI) was highest in the favorable oblique scenario (5.14), followed by the posterior (2.11) and centered plan (1.61), whereas the unfavorable oblique scenario showed the lowest value (0.76).

### Dose constraints in organs at risk

The comparative analysis showed that the esophagus was the organ most sensitive to catheter positional variation, with a marked increase in D0.035cc and D2% in the posterior configuration. In contrast, the centered and unfavorable oblique configurations showed intermediate values, while the favorable oblique orientation presented a moderate increase compared to the 2D plan.

In the right lung, greater dosimetric heterogeneity was observed, with the unfavorable oblique scenario showing the highest D0.035cc value. The left lung showed smaller variations and overall low doses across all scenarios.

Cardiac doses were low in all scenarios, with no clinically relevant differences. In the aorta, the favorable oblique plan showed the highest D0.035cc and D2% values, whereas the unfavorable oblique plan showed the lowest values, with minimal variation in mean and low doses (D50% and D98%).

## Discussion

HDR endotracheal/endobronchial brachytherapy offers advantages derived from its intraluminal nature, allowing the delivery of high doses with a steep dose gradient. This enables maximal dose deposition in the affected tracheal mucosa while limiting irradiation of critical organs at risk, such as the esophagus, spinal cord, and major vessels. This characteristic allows brachytherapy to represent a safe alternative to external beam radiotherapy techniques, avoiding the exposure of larger volumes of healthy tissue to low or intermediate doses^13^.

The present study compares, within the same clinical case, a real two-dimensional (2D) clinical HDR endotracheal brachytherapy plan with four simulated three-dimensional (3D) plans, modifying exclusively the spatial position of the catheter relative to the PTV. This approach allows assessment of the impact of applicator location on tumor coverage and dose distribution to organs at risk, which represents the main source of uncertainty in 2D planning.

Our results show that PTV coverage is highly dependent on the spatial relationship between the catheter and the target volume. The favorable oblique scenario, in which the catheter is positioned closer to the tumor, achieved the highest values of D90, D98, and V100. However, this improvement was accompanied by a marked increase in V150 and a high homogeneity index (HI), reflecting a highly heterogeneous dose distribution with large regions of overdose.

Conversely, when the catheter was positioned farther from the tumor (unfavorable oblique plan) or posteriorly, coverage decreased significantly, with relevant reductions in D90 and V100.

The centered 2D plan showed intermediate values, with nearly complete PTV coverage (V100: 99%), suggesting that in relatively concentric tracheal lesions with a properly centered catheter, conventional 2D planning may provide acceptable coverage (assuming the lack of anatomical confirmation by imaging).

The marked increase in V150 in the favorable oblique scenario highlights a clinically relevant issue: improved coverage does not necessarily imply better dosimetric quality. High-dose regions may increase the risk of local toxicity, particularly in structures such as the tracheal wall, potentially leading to necrosis, ulceration, or fistula formation.

One of the most relevant findings of this study is the significant variability in OAR dose depending on catheter position. The esophagus was the most sensitive organ to positional changes, particularly in the posterior scenario, where marked increases in D0.035cc and D2% were observed. This finding has clear clinical implications, given the potential risk of esophagitis, ulceration, or tracheoesophageal fistula in cases of overdosing^14^.

Similarly, variations in maximum doses were observed in the ipsilateral lung and aorta, particularly in oblique configurations. Although mean doses were low in this clinical case, these findings illustrate how 3D planning allows identification of potential risks that remain undetected in 2D planning due to the lack of OAR delineation.

Nevertheless, 2D planning remains a validated technique in resource-limited settings, given its speed and historically demonstrated effectiveness. The choice between both techniques should consider technological availability, the experience of the multidisciplinary team, and the anatomical characteristics of the case^15^.

It is important to note that in a real (non-simulated) 3D planning scenario, although the initial optimization would be based on prescription points located 1 cm from the catheter, the plan would subsequently be adapted to patient-specific anatomy and catheter position. This adaptation inherently aims to maximize target coverage while maintaining low doses to organs at risk.

This study presents limitations inherent to its design, including the use of a single clinical case and the fact that 3D plans were simulated rather than clinically applied. Nevertheless, the controlled comparative design clearly illustrates the dosimetric impact of catheter positioning.

## Conclusions

The results demonstrate that catheter location is a determining factor in endobronchial brachytherapy, significantly influencing both PTV coverage and dose delivered to organs at risk.

2D planning may provide acceptable coverage in favorable scenarios with a centered catheter. However, 3D planning offers more precise anatomical and dosimetric evaluation, allowing optimization of PTV coverage and identification of underdosage or potential overdosage in organs at risk.

These findings support the progressive implementation of three-dimensional planning as the standard of care^13^, particularly in lesions located near radiosensitive structures. Nevertheless, conventional 2D planning remains an effective alternative, especially in resource-limited settings or situations requiring rapid intervention.

Finally, despite the high prevalence of lung cancer, available studies on endobronchial brachytherapy are scarce and highly heterogeneous, often based on small series, which limits the ability to draw robust conclusions.

## Figures and Tables

**Figure 1:**
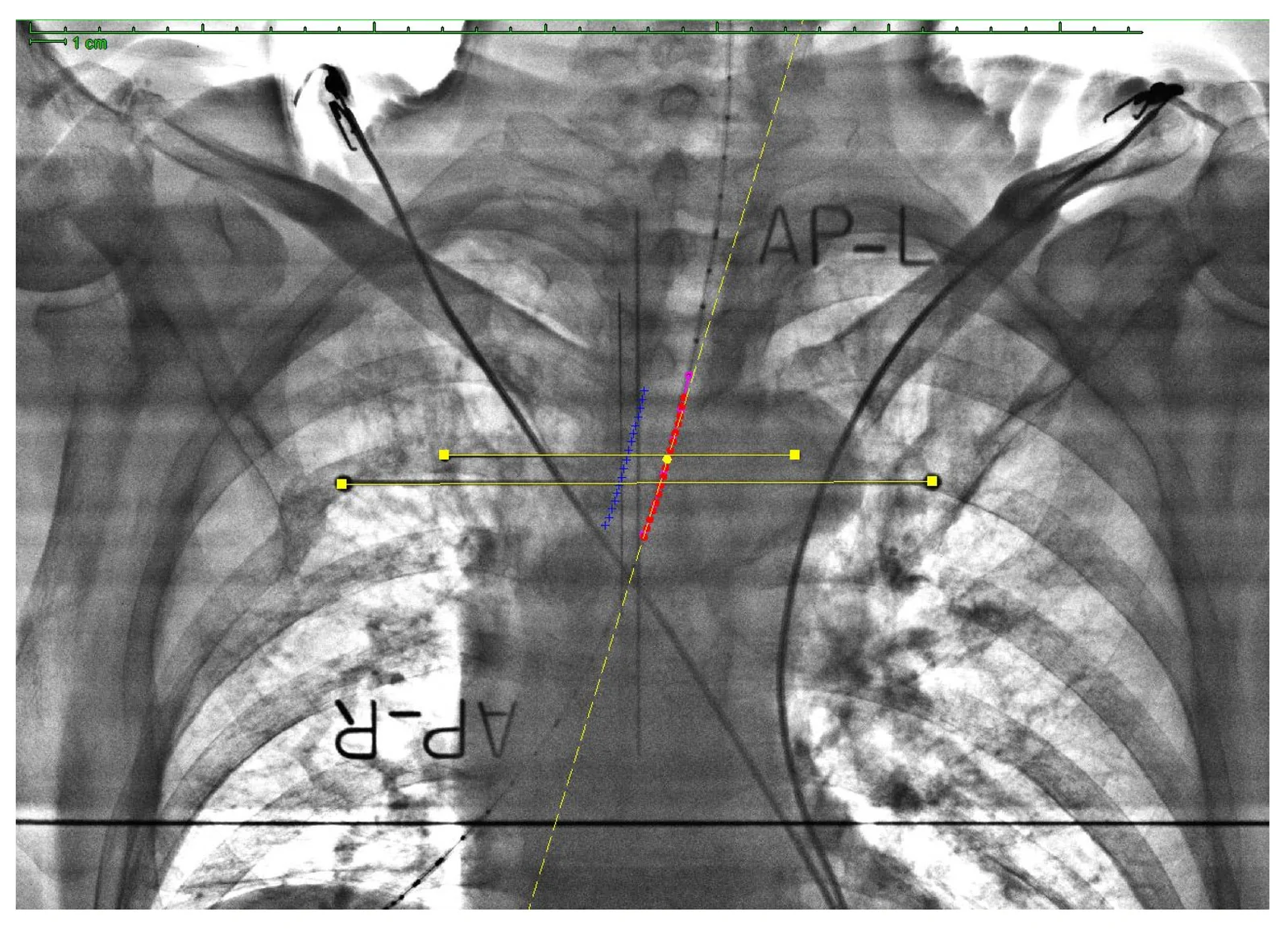
Anteroposterior chest radiograph with endobronchial catheter containing a simulation probe for 2D planning. Yellow indicates projected axes, pink the catheter reconstruction, red the source dwell (activation) positions, and blue the prescription points (1 cm from the catheter).

**Figure 2:**
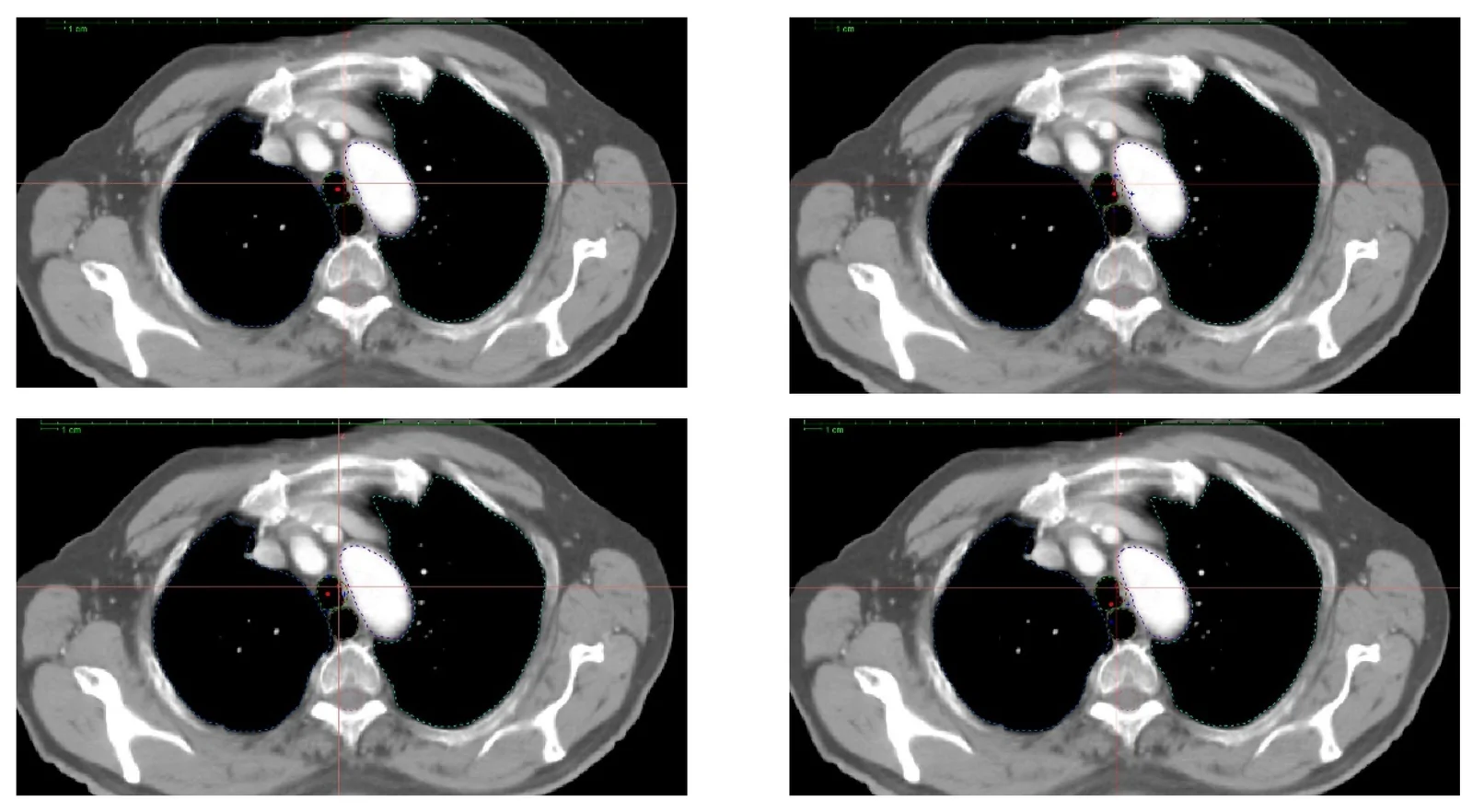
Axial image showing the four scenarios at the central PTV slice. Upper left: scenario A (centered catheter); upper right: scenario B (favorable oblique); lower left: scenario C (unfavorable oblique); lower right: scenario D (posterior catheter). The catheter is identified by the red point (source dwell position), and organs at risk are also delineated.

**Figure 3:**
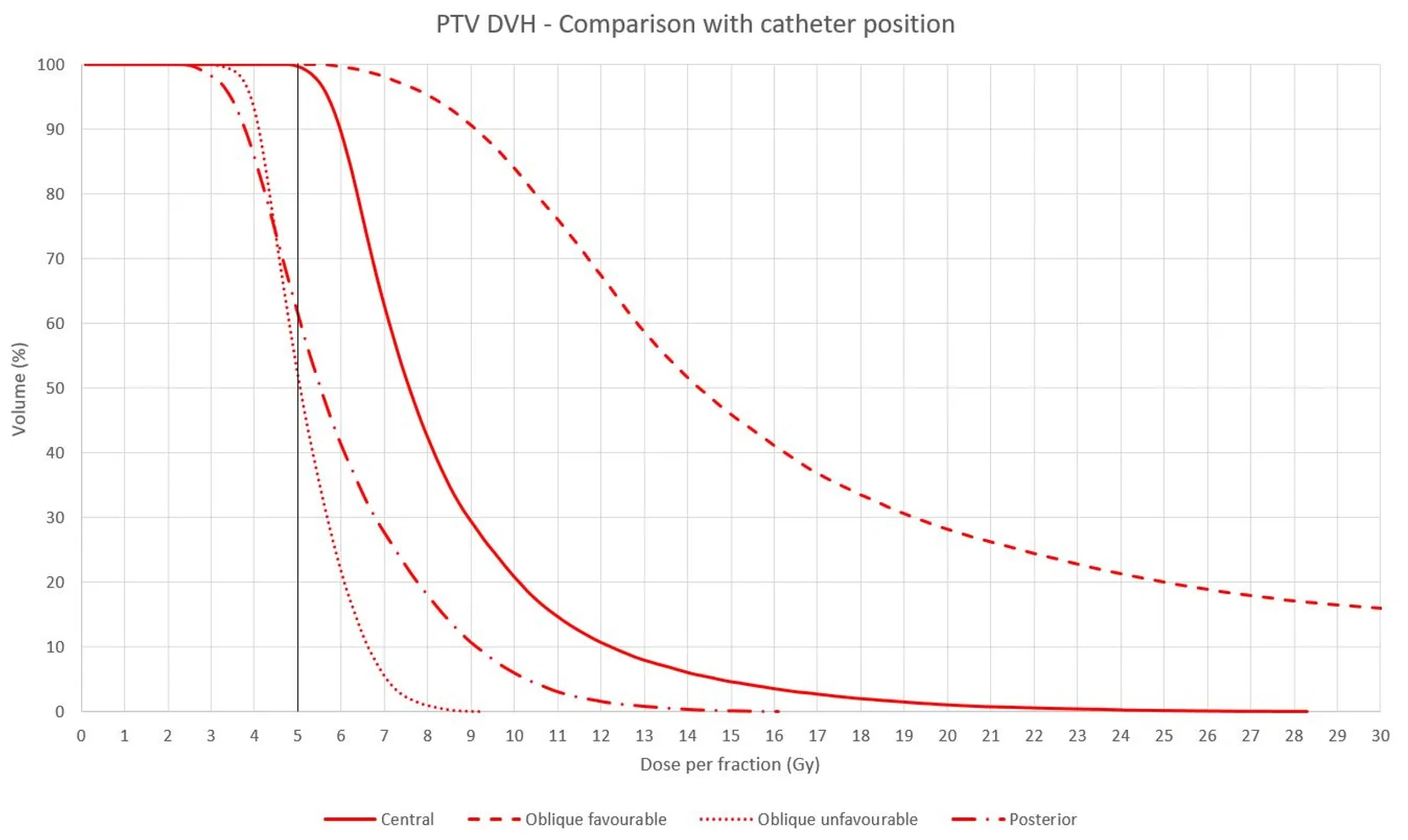
Dose-Volume Histogram (DVH) comparing the four plans for the PTV. The solid line represents scenario A, the dashed line scenario B, the dotted line scenario C, and the dash-dot line scenario D. The vertical black line indicates the prescribed dose of 5 Gy. This figure clearly shows differences in PTV coverage depending on catheter position.

**Figure 4:**
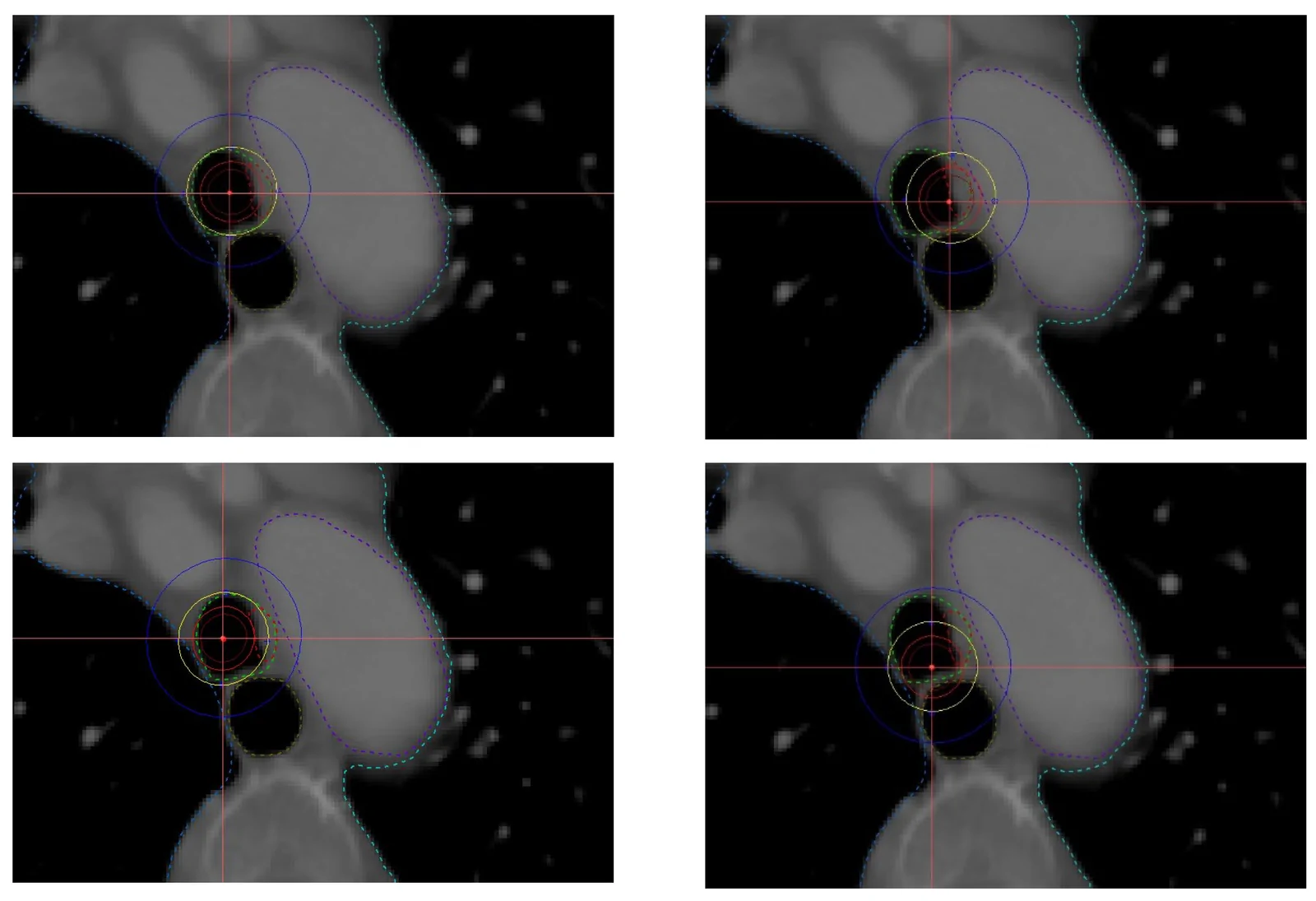
Axial isodose distribution for the different catheter configurations at the central PTV slice. The upper left image shows scenario A (centered catheter); upper right, scenario B (favorable oblique); lower left, scenario C (unfavorable oblique); and lower right, scenario D (posterior catheter). The catheter is identified by a cross marker. Isodose curves correspond to 10 Gy (maroon), 7.5 Gy (red), 5 Gy (yellow, prescription dose), and 2.5 Gy (blue). The PTV is contoured in red, located on the left side of the trachea (adjacent to the aorta, contoured in purple).

**Table 1:** Technical parameters of the evaluated plans:

Parameter	2D Plan	3D Centered	3D Favorable oblique	3D Unfavorable oblique	3D Posterior
Planning system	Oncentra Brachy	Oncentra Brachy	Oncentra Brachy	Oncentra Brachy	Oncentra Brachy
Source	Ir-192	-	-	-	-
Active length	4 cm	4 cm	4 cm	4 cm	4 cm
Prescription point	1 cm from catheter	1 cm from catheter	1 cm from catheter	1 cm from catheter	1 cm from catheter
Prescribed dose	5 Gy	5 Gy	5 Gy	5 Gy	5 Gy
Number of dwell positions	17	17	17	17	17

**Table 2:** Dosimetric parameters of the target volume (PTV) according to catheter position:

Parameter	Centered (2D Plan)	Favorable oblique	Unfavorable oblique	Posterior
D90 (Gy)	5,9564	9,0488	4,093	3,7752
D98 (Gy)	5,3938	6,9455	3,6849	3,0994
V100 (%)	99,63	100	51,4	61,29
V150 (%)	50,87	96,47	2,11	22,33
IH	1,61	5,14	0,76	2,11

**Table 3:** Dosage in organs at risk according to catheter position.

Organ	Parameter	Centered (2D Plan)	Favorable oblique	Unfavorable oblique	Posterior
Esophagus	D 0,035cc D2% D50% D98%	4,8848 3,8246 1,2324 0,1558	7,2653 4,5918 1,3032 0,1506	5,0979 3,384 1,1019 0,1613	15,6876 8,5517 1,6744 0,1532
Right lung	D 0,035cc D2% D50% D98%	5,0654 0,877 - -	3,1654 0,7608 - -	10,6309 0,9535 - -	6,2309 0,4357 - -
Left lung	D 0,035cc D2% D50% D98%	2,1967 0,4362 - -	2,8935 0,4748 - -	1,7967 0,4052 - -	2,0967 0,4357 - -
Heart	D 0,035cc D2% D50% D98%	0,6335 0,1499 - -	0,6002 0,1482 - -	0,6335 0,1551 - -	0,6002 0,1193 - -
Aorta	D 0,035cc D2% D50% D98%	5,1893 2,4229 0,3451 0,106	9,6125 3,0865 0,3521 0,1053	3,6104 1,9442 0,3311 0,1053	4,1156 2,081 0,3438 0,1037

## Data Availability

The data used to support the findings of the present study are available from the corresponding author upon request.
